# Hospital and Institutional News

**Published:** 1920-02-28

**Authors:** 


### HOSPITAL AND INSTITUTIONAL NEWS.

### DEATH OF MAJOR CHRISTIE.

The *Times* of Wednesday last contained an advertisement of the Reception on behalf of the appeal fund of the London Fever Hospital, Islington, at the Mansion House on Thursday, when Princess Mary attended. In this announcement the name of Major William Christie was mentioned as Officiating Secretary, and in the same issue of our contemporary the death of this veteran hospital officer was notified. The Fever Hospital had quite recently appointed, as Secretary, Major W. J. H. Brand, who, after a few months of service in that capacity, succumbed to heart failure following on an attack of scarlet fever (The Hospital, January 31, page 401). Major Christie returned to his old post as Secretary, which he had filled for thirty-three years, but unfortunately did not hold office for long; he died on Monday last at his residence at Clapham, at the advanced age of eighty-three. Major Christie, before he entered upon his hospital career, served in the 13th Hussars (Light Dragoons). His age will come as a surprise to many who knew him, for his appearance and vigour were more suited to one at least twenty years younger.

### POST-GRADUATE DEVELOPMENTS.

The latest suggestion for the much-needed improvement of post-graduate medical teaching in London is that the Post-Graduate Fellowship of Medicine should take over St. George's Hospital as the Central Post-Graduate School for London. There is a certain attractiveness about the suggestion, several of the advantages of which are obvious enough. Thus St. George's Hospital has a sufficient number of beds for a really first-rate post-graduate school. It is centrally situated and accessible, a very important matter for post-graduate students. It has various amenities—such as club-rooms, residents' quarters, clinical laboratories—designed on the scale necessary for a teaching hospital as opposed to a non-teaching one. It has a first-rate staff, and long traditions, so that there is much to be said for this particular proposal. The need for getting London recognised as a leading post-graduate centre is urgent. The materials are all there; the teachers are second to none, the mass of clinical opportunities is unrivalled—only the organisation (and the students) are required. A correspondent writing recently from South Africa emphasised this very strongly. He said that all the keenest young Dutch doctors, trained in South Africa, come over to Europe for post-graduate study before starting in practice. Before the war they went to Germany and Austria; now they are going to Holland, where they imbibe (so he says) anti-British propaganda more freely and more disastrously than ever they did in Germany. So the problem has an Imperial as well as a purely medical aspect. Incidentally, there have been persistent rumours lately of a projected amalgamation between St. George's and St. Thomas's; such a scheme would rather "knock on the head" the Fellowship of Medicine's interesting scheme.

### MORE SUGGESTIONS FOR RAISING MONEY.

Manchester is one of the active centres in the financial campaign which the voluntary hospitals are undertaking. Sir William Milligan lately said that the problem would be solved if every person in Manchester would subscribe a penny a week. This well-tried suggestion gains additional interest from the letter which it evoked from the chairman of the Hospital Saturday Fund in Manchester. Thanking Sir William Milligan for his suggestion, the chairman said that it was the doctrine which the Saturday Fund had been preaching for years. It had raised about £5,000 a year, and would have raised more had it not been for "the apathy of employers and employed." The limits of this success are a striking illustration of an argument which The Hospital has emphasised for years—namely, the need for the organisation of sectional subscriptions from every class in the community. The adoption of this plan at North Staffordshire a year or so ago produced astonishing results. Since then the Leeds General Infirmary has drafted a most carefully considered plan for an Employers' Hospital Subscription Fund. It has been very well received. Why should not such a fund be developed on a wide scale at Manchester? Would the Saturday Fund then also develop a scheme for the collection of weekly pennies from other classes besides the men in workshops and factories?

### NEARLY £100,000 FOR A CHARITY.

The will of Major Thomas Bott, late 6th Dragoon Guards, of Hove, Sussex, must set up almost a record in the number of charities mentioned. Major Bott, who died in his ninetieth year, was a Crimean and Indian Mutiny veteran. He left estate valued at £152,391 and, after making provision for relatives and servants, left £1,200 to the National Lifeboat Institution and £200 each to the Dogs' Home, R.S.P.C.A., Dr. Barnardo's Homes, and the Royal Free Hospital. These charities, or some of them, are also mentioned in the directions as to the division of the residue of the estate, which is left to quite a number of institutions, the classes prominently mentioned being the Blind and Societies for the protection of animals. The hospitals included are: The London, Star and Garter, Seamen's, St. Bartholomew's, St. Thomas's, Charing Cross, Guy's, King's College, the Royal Free, St. George's, St. Mary's, and the Cancer.

### PUBLIC DEBATE ON THE VOLUNTARY SYSTEM.

The Beckett Hospital, Barnsley, as we have often noted, benefits during the summer from the sports and meetings which are traditionally held in the district. The hospital festival in fact is a regular institution in parts of Yorkshire. Addresses are given, and in one of these last year Councillor Joyce strongly advocated the State control of hospitals. Mr. A. Whithorn, chairman of the Beckett Hospital, thereupon invited Councillor Joyce to debate the question in public. The offer was accepted some months later, and an interesting discussion was held. Mr. Joyce was a worthy opponent because he spared his audience the crude arguments which are sometimes put forward on his side, and endeavoured to show that State control was possible without red-tape. Mr. Whitham, who has forty years' experience of the voluntary system behind him, enlarged upon the antiquity, the efficiency, and the expediency of that system, which, he pointed out, had lasted in England since 1070. The debate showed a keenness, a lively interest in the hospital question, a conviction, which suggests that the doctrine of personal service is an active force in Barnsley.

### THE LEEDS SCHEME AT ABERDEEN: A PENNY A WEEK FROM EMPLOYEES.

The Leeds scheme for an Employers' Subscription Fund is proving an infectious example, even if we regard it merely to be the most carefully considered expression of a general tendency. Thus Mr. George Davidson, chairman of Aberdeen Royal Infirmary, who has long encouraged collections among both employers and their men, now urges the former to assert themselves voluntarily according to the number of the men upon their wage-list. The base which he proposes is that of 1d. per week per man, or 8s. 4d. a week per hundred workmen, a figure which should be compared with that mentioned in the Leeds scheme, which we published in The Hospital of Feb. 7, p. 437. Mr. Davidson thinks that this might come to be regarded as a minimum, if only because the men in many industries now voluntarily give more than their original weekly penny. With the employers, however, a beginning in many places has yet to be made, and perhaps it is well to begin with the same subscription as that adopted by the men when workers' funds were first established. In the face of such schemes as that begun at Leeds, and about to be begun at Aberdeen, there is no need to waste breath upon "alternatives." Mr. Davidson, however, lately put the alternatives very well: which do you prefer, he asked, regular voluntary contributions or regular compulsory rates?

### A NEGATIVE POLICY AT GLASGOW.

We hear that the speeches made at the recent annual meeting of the Glasgow Royal Infirmary show divided counsels in the minds of the managers. Mr. Timothy Warren, the hon. treasurer, is reported to have said that the Royal Infirmary not only could not, but should not receive paying patients; he also hinted a doubt whether weekly collections from the works did not savour of compulsion, though on the whole he did not think they did. He further is reported to have said that there could be no efficient half-way house between the voluntary and the "full-fledged State hospital." Another speaker was opposed to subscriptions from firms, and thought a personal subscription from each director preferable. Our impression on reading these statements is one of keen discovery that responsible hospital managers, at the present financial crisis, and when speaking on behalf of the acute needs of their own great hospital, should be so deeply confused in mind, so halting in suggestion, so abysmally lacking (as it seems to us) in the A B C of hospital policy. We record the remarks as an example of what not to say on these occasions. A Note is not the place to repeat again the principles which The Hospital has so often argued in respect to the pay-ward, the grant-in-aid for special services, the Employers' Subscription Fund, the three points so sadly mishandled at Glasgow. But so long as a hospital's policy is ruled by this confusion of ideas, so long will there be no escape from debt for the unfortunate institution.

### HOSPITALS AND SPORTING CHANCES.

The question of the legitimacy or otherwise of this or that source of hospital income is not always a matter of principle. It depends, within obvious limits, on the local feeling of the district which a hospital serves. Thus while tombolas are taboo in East Anglia, "a draw" is considered decorous in the West. We see that one was held in favour of the shilling fund of a county infirmary, for which one enthusiast alone sold nearly one hundred and fifty tickets. Forty-four prizes were given, apparently not in cash, and the expenses were met privately. The sum raised was not large; the hospital is not a very big one; but the result was considered to justify the draw, to which no one offered any objection. Those who favour this form of raising money would do something to allay prejudice against it, if they themselves took advantage of such lotteries (a convenient name) as do occur, like the big one for the War Loan whose winners were announced last week, by announcing that they were taking tickets on behalf of their local hospital. In the event of such a ticket winning one of the better prizes, we believe there would be considerable local enthusiasm, and the mere fact that the hospital had been allowed to benefit by the chance of fortune, through the generosity of one of its subscribers, would do much to place the good side of sporting chances in a favourable light. It would be essential to announce beforehand that the hospital was concerned in the result, to distinguish any sum which might accrue from the casual gift of an ordinary donor.

### AGAINST MUNICIPAL HOSPITALS.

Mr. George E. Priestman, who received a presentation on retiring from the chairmanship of the House Committee of the Bradford Royal Infirmary—an institution with which he has been most honourably connected for very many years—sees a bad time ahead for the ratepayers should voluntary hospitals cease to exist. "I am dead against municipal hospitals," he said, "they are more expensive and would not be so well or so economically managed. Unless they are very careful, the expense would run to at least a shilling, and perhaps two shillings, on the rates." The Lord Mayor expressed his opinion that a new Bradford Hospital, which is so badly needed, would appeal to all sensible people as the best form of war memorial.

### PATIENTS WHO DISCHARGE THEMSELVES.

The London Insurance Committee appointed a committee to consider as to the reasons why some insured persons discharge themselves from sanatoria before the completion of their treatment, and as to the steps which should be taken to prevent such early discharges. This committee now gives the following reasons:—(1) Disinclination for the necessary discipline and curtailment of personal liberty. (2) Difficulties at home. These for the most part are financial in character, but social and family problems are not infrequently the cause of discharge. (3) During the colder seasons of the year, dislike of sanatorium life, which, under the best circumstances, is somewhat uncomfortable and often rigorous. (4) Disappointment at want of progress. This is common among the more advanced cases. Complaint is rarely made as to the food supplied at various institutions. Since the rationing order came into force, sanatoriums have worked to a scale laid down by the Ministry of Health. Lately, some complaint has been made of the food at two institutions. In both cases enquiries have been instituted.

### THE PERSONAL TOUCH AND THE PATIENT.

Suggestions for the prevention of early discharge are:—(1) The better education of patients as to the nature of consumption and the method of its treatment and cure. It is an experience that the educated classes, being better informed, and realising more fully what is at stake, are more ready to put up with discomforts and to make immediate sacrifices in the hope of gaining ultimately thereby. Simply expressed addresses to patients are of assistance. It should be the duty of a Sanatorium Superintendent to see that every individual patient has his position explained to him by a responsible officer. It goes without saying that personal and sympathetic relations must exist between patients and officers if the former are to be influenced favourably. (2) The average duration of stay is longest in the institutions at which most pains are taken to interest patients, in short in which there exists a personal touch, as compared with a mechanical, somewhat impersonal routine. (3) Financial assistance to dependants. This is regarded of great importance. (4) In some institutions there is room for an increased mitigation of the rigours of the winter. It must be remembered that sanatoriums are designed for the treatment of early cases, and that owing to the lack of other accommodation many cases of advanced disease are sent to those institutions. So long as this continues, self-discharge among the more advanced cases will be numerous. Patients enfeebled by long-standing disease and unable to exercise freely have not the vitality or recuperative powers to withstand the conditions of sanatorium life during the winter, except, perhaps, at institutions in sheltered positions on the South Coast.

### WIGAN'S BELIEF IN THE VOLUNTARY SYSTEM.

The Royal Albert Edward Infirmary, Wigan, receives from the workers some £10,000 per annum, mainly the result of a fixed contribution of 1d. per week. Wigan's hospital, however, is not content with this, and an appeal was recently made by Mr. H. Lucas, the general superintendent and secretary, for 2d. per week. At the first colliery to consider this a ballot was taken, and the following interesting figures resulted:—*For* 674; against 149. Wigan is a district where so much has already been accomplished that one might expect a reaction, and to hear the cry of "nationalisation." Other collieries, works, etc., have responded, and the general adoption of the additional 1d. would seem to be well in sight. A movement is afoot whereby the whole of the tradesmen and their employees will contribute regularly through the medium of the local Chamber of Trade. Mr. Lucas has also had gratifying results from appeals for new subscribers and donors. The assistance of the Wigan and District Division of the British Red Cross Society has been won, and recently it gave the splendid sum (almost the whole of the balance of its war funds) of £2,000 for the transformation of a day-room into an up-to-date ward for pensioner sailors and soldiers. This will be known as the "Red Cross" Ward.

### OPEN-AIR MARKETS AND PUBLIC HEALTH.

The many open-air markets recently permitted in order to combat the profiteering retailer have probably had excellent results in that direction; but have they endangered the public health by the exposure of the food? The sale of butcher's meat, bacon, fish, rabbits, etc., which undergoes cooking before consumption, provided that it is fit for food offers no special harbour to disease carriers. The same applies to articles of household use which are to be found on the stalls, such as soap, polish, and brushes. But those articles of food which are eaten without any cooking—for example, grapes, sweets, and cakes—the condition of these can be imagined after an exposure for several hours on a windy day, when they may be covered with dust, road sweepings, and particles of manure! Is the condition of these stalls in a crowded district considered in relation to the local spread of an infectious disease, or are the stalls beneath the dignity of the sanitary inspector?

### INTEMPERANCE IN INDIA.

The extent to which intemperance prevails in certain parts of India is hardly realised in England. Recently special measures have been resorted to, with the object of combating intemperance in Assam. The consumption of country liquor in the colliery districts has also been engaging the attention of the Government of Bihar and Orissa. Some improvements have been effected by reducing the quantity of spirit which any person may have in his possession without a pass, and by increasing the price. The opening of coffee shops and also tea shops near the country liquor shops as a counter-attraction, which has been tried in various parts of the country, has failed, and many temperance establishments have had to close for want of customers. During the past few years sections of Indian communities, previously temperate, have taken to alcohol. The commencement of the previous wave of excessive drinking is traced back to the recent influenza epidemic, during which the aboriginals commenced drinking under the impression that country spirit was a prophylactic against the disease. The practice is not confined to one province, but is prevalent in all parts of India.

### PRIVATE PATIENTS AT PENZANCE.

So great has been the pressure on the beds of Penzance Hospital that the board room has had to be taken for the nursing staff, and the latter's room turned into a private ward. In this way twenty-nine beds have been provided. It is interesting to note that at Penzance also the increased demand has come mainly from private patients; there is still needed another ward for them. Rebuilding is now a necessity, and an appeal is being issued for £12,000 to build and to maintain a new children's ward.

### A LESSON FROM THE SATURDAY FUND.

Those doubters who have confessed a want of faith in the future of the voluntary system would do well to meditate on the achievement of the Hospital Saturday Fund. Its contribution for 1919 was over £4,000 above the total of the previous year. The fact is known, but its significance is missed. The rise took place during the change from war to peace, when many munition factories were shut or closing—when, that is to say, their contributions were being either reduced or stopped. That is remarkable. The reason is stated to be that the loss felt in this quarter was more than counterbalanced by the efforts of the ten thousand honorary secretaries, who returned from the Colours to resume their pre-war work of collecting for the Saturday Fund, in the workshops and warehouses. To test this claim to the utmost we will wait yet one year more, but that the increase should have been attributed to this cause rather than another is most significant. The fact should put the most timid of hospital Thomases to shame.

### THEFT FROM AN OPERATING-THEATRE.

The post-war epidemic of crime and theft has attacked even hospitals. A curious case recently occurred at Nottingham, where a porter at the General Hospital confessed in Court to stealing two Treasury notes from the pocket of one of the surgeons, Mr. C. H. Allen. During operations, according to the evidence, thefts have repeatedly occurred of late from the coats of the surgeons in the theatre ante-room. It was through the number of a note being taken that the porter was detected

### THE DOCTOR'S DEBT TO HOSPITALS.

Dr. Francis was lately presented with a silver salver on his retirement after thirty years' service on the staff of the Birkenhead and Wirral Children's Hospital. In acknowledging the gift, Dr. Francis said, that a doctor on a hospital staff "gets far more than he gives," for the tremendous experience and opportunity of studying cases was of enormous value to him. True as this is, it is not always admitted, and Dr. Francis's sincerity is to be honoured accordingly.

### SANATORIUM RESULTS IN BIRMINGHAM.

An inquiry is to be held into the results of sanatorium treatment in Birmingham; the report will be presented to the Insurance Committee. A stimulus was given to the proposal by the statement of Mr. H. C. Teall, who said that, according to the report of the medical adviser to the Committee, no fewer than 70 per cent. of the insured persons who were recommended for sanatorium treatment in London during 1914 died before the end of 1918. He believes that the condition of the same class of people in Birmingham is much less tragic. But the need for an inquiry is obvious.

### HOSPITALS AND LOCAL AMBULANCES.

The Keighley Town Council has decided that the ambulance may be used free of charge for accidents within the borough, and that a minimum charge of five shillings shall be made to convey medical or emergency cases to any institution in the borough; the rate, if levied, is to be 1s. 6d. per mile to any institution outside. Infectious cases and the dead are not eligible for conveyance. The entire area is limited by the district served by the Keighley Victoria District Hospital. The subscribers wish that the ambulances shall form part of the hospital service, and be as free as the other services connected with the hospital.

### NORTH ORMESBY HOSPITAL.

A certain edge is given to the appeal for £70,000 on behalf of North Ormesby Hospital by a list of the things which the hospital is without. There is no massage and electrical department, there are no medical wards for men and women, no hospital laundry, no second theatre, and no suitable nurses quarters. It is a formidable list, and as North Ormesby claims to be the centre of the iron industry and to serve the Cleveland district of the North Riding, there ought to be no difficulty in raising the sum. Mr. Stanley Brunt, the Secretary, is enlisting collectors and helpers, and we commend to his attention and to that of his committee the details of the new scheme for an Employers' Subscription Fund at Leeds, which we published a week or two ago.

### THIS WEEK'S DRUG MARKET.

The upward movement of prices is still the principal feature of the market, and so long as the heavy export demand continues we may expect this feature to remain. Quinine is dearer and in active demand. The result of heavy buying of citric acid is that supplies are now scarce and prices much higher. The little remaining supply of ergot of rye is offered at dearer rates. Ipecacuanha still moves upwards in price. Cream of tartar and citric acid are dearer. Gentian root and gamboge are scarce. Resorcin is still difficult to find. Prices of aspirin, phenacetin, and phenazone are firmly maintained.

